# The virulent, emerging genotype B of *Deformed wing virus* is closely linked to overwinter honeybee worker loss

**DOI:** 10.1038/s41598-017-05596-3

**Published:** 2017-07-12

**Authors:** Myrsini E. Natsopoulou, Dino P. McMahon, Vincent Doublet, Eva Frey, Peter Rosenkranz, Robert J. Paxton

**Affiliations:** 1 Institute for Biology, Martin-Luther-University Halle-Wittenberg, Hoher Weg 8, 06120 Halle (Saale), Germany; 2 School of Biological Sciences, MBC, Queen’s University Belfast, Belfast, BT9 7BL UK; 3 Institute of Biology, Free University Berlin, Schwendenerstr. 1, 14195 Berlin, Germany; 4 Department for Materials and Environment, BAM Federal Institute for Materials Research and Testing, Unter den Eichen 87, 12205 Berlin, Germany; 5 German Centre for Integrative Biodiversity Research Halle-Jena-Leipzig (iDiv), Deutscher Platz 5e, 04103 Leipzig, Germany; 6 Apicultural State Institute, University of Hohenheim, 70599 Stuttgart, Germany; 7 Section for Organismal Biology, Department of Plant and Environmental Sciences, University of Copenhagen, Thorvaldsensvej 40, Frederiksberg, Denmark; 8 Centre for Ecology and Conservation, University of Exeter, Penryn, UK

## Abstract

Bees are considered to be threatened globally, with severe overwinter losses of the most important commercial pollinator, the Western honeybee, a major concern in the Northern Hemisphere. Emerging infectious diseases have risen to prominence due to their temporal correlation with colony losses. Among these is *Deformed wing virus* (DWV), which has been frequently linked to colony mortality. We now provide evidence of a strong statistical association between overwintering colony decline in the field and the presence of DWV genotype-B (DWV-B), a genetic variant of DWV that has recently been shown to be more virulent than the original DWV genotype-A. We link the prevalence of DWV-B directly to a quantitative measure of overwinter decline (workforce mortality) of honeybee colonies in the field. We demonstrate that increased prevalence of virus infection in individual bees is associated with higher overwinter mortality. We also observed a substantial reduction of infected colonies in the spring, suggesting that virus-infected individuals had died during the winter. Our findings demonstrate that DWV-B, plus possible A/B recombinants exhibiting DWV-B at PCR primer binding sites, may be a major cause of elevated overwinter honeybee loss. Its potential emergence in naïve populations of bees may have far-reaching ecological and economic impacts.

## Introduction

Insect pollination, which is carried out mostly by bees, is required for 75% of all food crops^1^; the global value of insect pollination in 2005 has been estimated at €153 billion^2^, although this is likely to be an underestimate^3^. In addition, 85% of wild plants are pollinated by animals^4^, chief among these being bees. Bees are therefore of considerable economic and ecological importance. The honeybee *Apis mellifera* is by far the most important commercial pollinator and is relied upon heavily for the successful pollination of many food crops^5^. However, over the last decade severe yearly losses of honeybees in the Northern Hemisphere^6–9^ have raised concerns about food security^10^.

The emergence of several infectious diseases has coincided with elevated honeybee colony losses^11–13^. Notable among these are positive single stranded RNA (positive ssRNA) viruses that have risen to prominence since the arrival of a novel biological vector from Asia, the ectoparasitic mite *Varroa destructor*. The mite feeds on the hemolymph of honey bee pupae and adults^14^. Its ability to act as a viral vector and potential incubator of several honeybee RNA viruses has given rise to a new viral transmission route, thereby aiding the spread and re-emergence of several bee viruses^15–17^.

A major source of honey bee decline is colony depopulation during the winter. Colonies suffering elevated winter worker losses are more prone to collapse, resulting in overwinter colony losses (OCL)^18^. High rates of OCL are a continuing concern in temperate zones, both in Europe and North America, where 15–20% and 25–30% of colonies respectively have been consistently lost overwinter in recent years^19–22^. Pathogens may be major contributors to OCL. Among those that have been implicated in colony decline are several positive ssRNA viruses: *Kashmir bee virus* (KBV)^12^; *Slow bee paralysis virus* (SBPV)^23^; *Israeli acute paralysis virus* (IAPV)^11^; *Acute bee paralysis virus* (ABPV)^24, 25^; *Deformed wing virus* (DWV)^12, 24–26^; microsporidia, particularly *Nosema ceranae*, an emerging gut parasite from Asia^13, 27^; and *V. destructor*, due to its association with several of the viruses^28^.

One virus in particular, DWV, is considered an important predictor of colony failure^25, 26, 28–30^. *Varroa destructor* has altered the dynamics of DWV dramatically, reducing within-host genetic DWV strain diversity, and leading to the global spread and dominance of genetic variants related to DWV genotype A (DWV-A)^17^. We have recently shown that another recently described genotypic variant of DWV, genotype B (DWV-B, also known as *Varroa destructor virus-1* or VDV-1^17, 31, 32^, is more virulent than classic DWV-A^33^. Although DWV-B has been recorded in Europe, Africa and Asia^34–37^ and is widespread in UK^33^, its role in OCL is currently unknown.

To date, field studies on the role of pathogens in OCL have been largely based on correlations between pathogens discovered at the colony-level and qualitative assessments of colony health (i.e. stable or collapsed colony). Quantitative field measures of intra-colony disease dynamics provide a better insight into their role in OCL. For example, measurements of pathogen prevalence and workforce mortality within colonies would permit a more precise understanding of the change in disease and overall health status of overwintering colonies. We address this shortfall by assessing the pathogen landscape in 28 colonies in two apiaries before and after the winter season while experimentally controlling *V. destructor* infestation levels. We then determined whether there is an explicit association between DWV-B and overwinter reduction of the honeybee colony workforce.

## Results

### Pathogen detection

To identify pathogens that may be responsible for overwinter honeybee mortality, we conducted an apiary study to assess the overwinter change in pathogen composition in 28 managed honeybee colonies. Sampling was performed once in autumn, at the end of brood rearing, and once the following spring, before commencement of major brood rearing, allowing us to exclude birth rate and directly to estimate loss of workers per colony. We initially used multiple ligation probe amplification (MLPA) to target a wide range of RNA viruses and RT-PCR to identify two microsporidian gut pathogens: *Nosema apis* and *Nosema ceranae*. For these analyses, we pooled 30 honeybee workers per colony and analysed one total RNA extract per colony.

Apart from chronic bee paralysis virus (CBPV), which occurred rarely in both autumn and spring, only viruses belonging to the DWV complex and *Black queen cell virus* (BQCV) could be detected by MLPA (Supplementary Table S1). We subsequently employed qRT-PCR on the same pooled samples to gain a more accurate understanding of the two prominent viruses: BQCV and DWV. In addition to BQCV and the original DWV-A genotype, we screened for DWV-B which shares 84% nucleotide identity with DWV-A^14^. DWV-A and -B were screened for by genotype-specific qRT-PCR amplification of Leader polypeptide (Lp) and RNA-dependent RNA polymerase (RdRp) genes; these are located at the 5′ and 3′ parts of the viral genome, respectively^14^.

**Figure 1:**
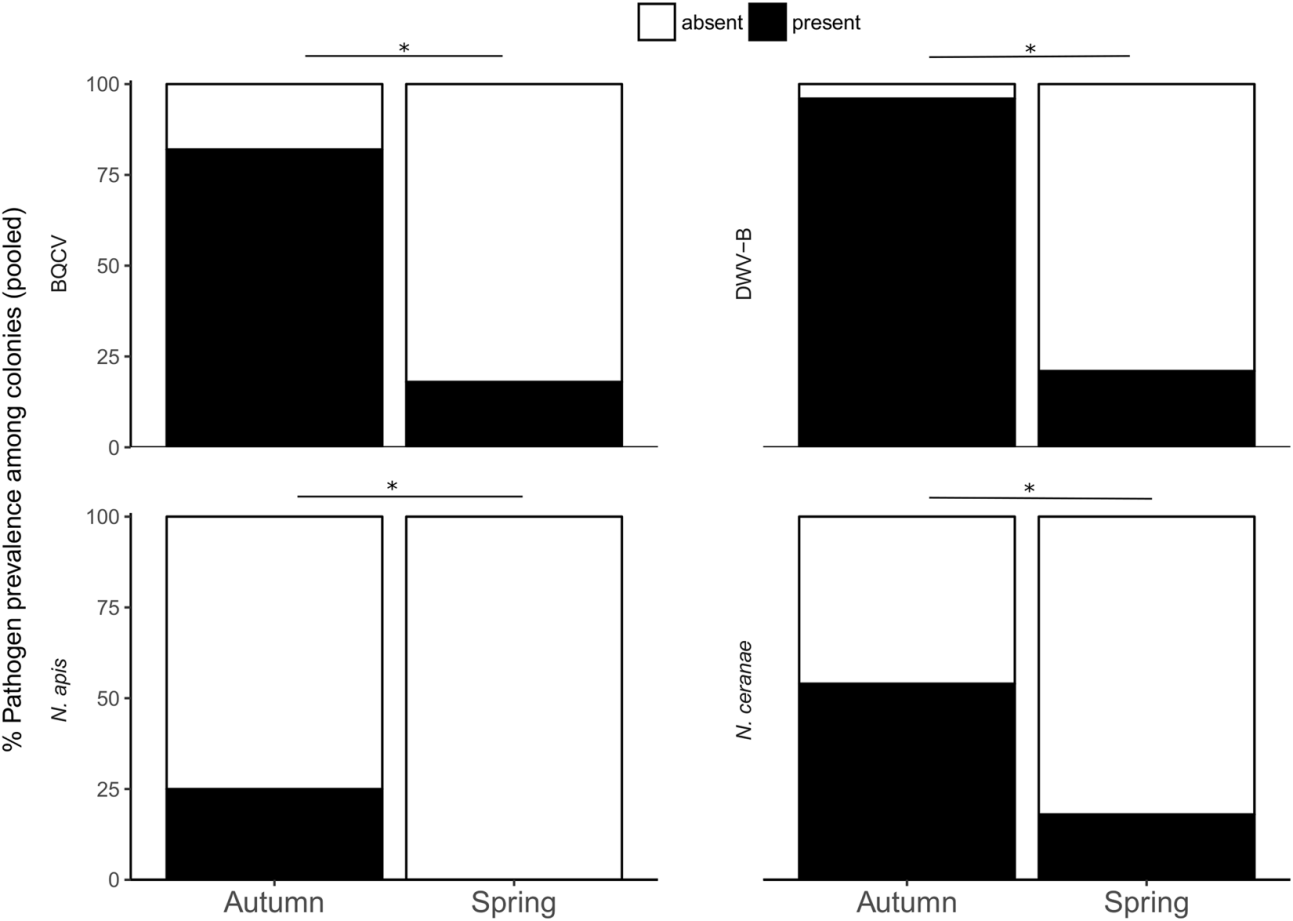
Proportional overwinter change in pathogen prevalence among colonies (qPCR screen of pooled worker samples, N = 30 bees per colony). Black = pathogen present, white = pathogen absent. *Significant comparisons: paired McNemar’s χ^2^ test p < 0.05.

**Figure 2:**
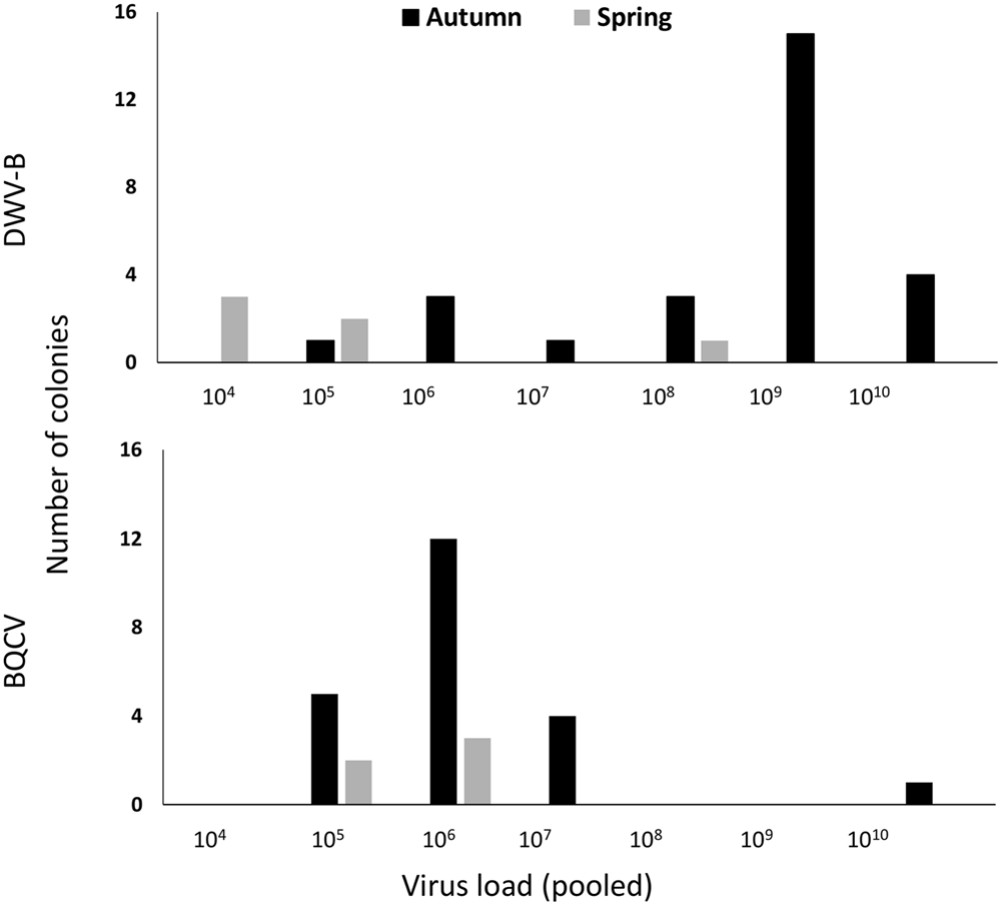
Histogram of DWV-B and BQCV viral titre in pooled colony samples (N = 30 bees per colony). Values represent average titre per bee. Black: viral titres in autumn; grey: viral titres in spring.

We found that 96% (N = 27) of colonies in the autumn contained significant quantities of DWV-B (Supplementary Table S2). Compared with the autumn, prevalence of DWV-B in spring colonies was significantly reduced, with 21% (N = 6) of colonies containing DWV-B (χ^2^_1_ = 19.1, p < 0.0001; Fig. 1). Some colonies in both autumn and spring contained very low viral loads of the DWV-A genotype, but these fell below an *a priori* detection threshold, and were only detected using the RdRp primer pair (Supplementary Table S2). This indicated that DWV-A was not (or at best, very rarely) a cause of infection in these colonies. DWV-B titres were also significantly reduced in spring colonies compared to autumn levels (paired Wilcoxon signed rank test V = 378, p < 0.00001) (Fig. 2). Potential caveats are that we may have missed very low titres of DWV-A because of pooling 30 bees per extract and that the qPCR primers for DWV-A could have been of lower sensitivity than those for DWV-B. To address the first caveat, we subsequently sampled individual honeybees (15 individuals per colony) but still did not detect DWV-A (see ‘*Overwinter mortality and pathogen prevalence*’). In relation to the second caveat, primer sensitivity was similar for DWV-A and DWV-B primers (Supplementary Table S4).

Interestingly, no recombinant viruses were detected (i.e. samples containing a mix of DWV-A and DWV-B fragments, Supplementary Table S2). However, a few samples positive for DWV-B in the RdRp region (N = 3 in autumn and N = 5 in spring) could not be amplified in the Lp region (Supplementary Table S2). This was attributed to the low amount of DWV-B detected coupled with the lower sensitivity of the DWV-B primer in this region (Supplementary Table S4).

The prevalence of BQCV was also high in autumn colonies (82%, N = 22; Supplementary Table S2), and then significantly reduced in spring to 18% (N = 5; χ^2^_1_ = 15.1, p = 0.0001; Fig. 1). BQCV titres were significantly lower in spring compared to autumn colonies (V = 272, p < 0.00001), also following the same pattern as for DWV-B. However, DWV-B titres in autumn sampling were significantly higher than those of BQCV (V = 377, p < 0.00001; see Fig. 2).

The Microsporidia *N. ceranae* and *N. apis* were detected in 54% and 25% of colonies in the autumn, respectively. Prevalences were also significantly lower in the spring, with 18% (N = 5) and 0% of colonies carrying each pathogen, respectively (χ^2^_1_ = 8.1, 5.1; p = 0.004, 0.023 respectively; Fig. 1).

### Overwinter mortality and pathogen prevalence

To understand the link between pathogens and overwinter workforce mortality in finer detail, we estimated the prevalence of pathogens within colonies by sampling 15 additional individual honeybees from each colony in autumn, using MLPA for RNA viruses and RT-PCR for *Nosema* spp. To verify the rarity of DWV-A across the colonies in the pooled samples (0% true prevalence from 30 pooled individuals per colony (N = 840), 95% confidence intervals (CIs): 0–0.45%), a subset of 37 individual workers found positive for DWV in MLPA (representing ca. 37% of DWV MLPA-positive samples, distributed randomly across 7 colonies) was subjected to qRT-PCR using the DWV-A specific primers. DWV-A was not present in any of the samples tested, supporting our previous analyses and suggesting that, if present, DWV-A had an extremely low titre. Therefore, DWV-positive individual honeybees screened by MLPA are treated as DWV-B, and are henceforth reported as such.

The analysis of individual honeybees confirmed the disease patterns detected in pools of 30 worker honeybees per colony (above), with DWV-B featuring most frequently among autumn bees (Supplementary Table S2). Generalized linear models (GLMs) were conducted to infer whether within-colony pathogen prevalence (including *V. destructor* infestation estimated from 150 honeybees per colony) were important predictors of overwinter workforce mortality. GLMs were conducted either with DWV-B prevalence (model 1) or *V. destructor* infestation (model 2) in addition to all other detected pathogens and apiary site as explanatory variables. Simplified final models either contained only DWV-B or only *V. destructor* but not BQCV as significant predictors of overwinter colony workforce mortality (Table 1).

**Table 1:** Best models explaining overwinter colony workforce mortality using GLMs and QAICc for model selection.

Response (Model)	Model	Parameters	Estimate	SE	*t*-value	*P*-value
Mortality	1	Intercept	−0.384	0.133	−2.893	0.033*
		DWV-B	0.297	0.132	2.257	
	2	Intercept	−0.401	0.125	−3.203	0.006*
		*V. destructor*	0.348	0.116	3.001	

Model 1: DWV-B as explanatory variable (*r* ^*2*^ = 0.16), Model 2: *V. destructor* infestation as explanatory variable (*r* ^*2*^ = 0.25). Viral and *Nosema* spp. prevalence was based on 15 individuals per colony while *V. destructor* infestation was estimated on a sample of 150 individuals per colony.

**Figure 3:**
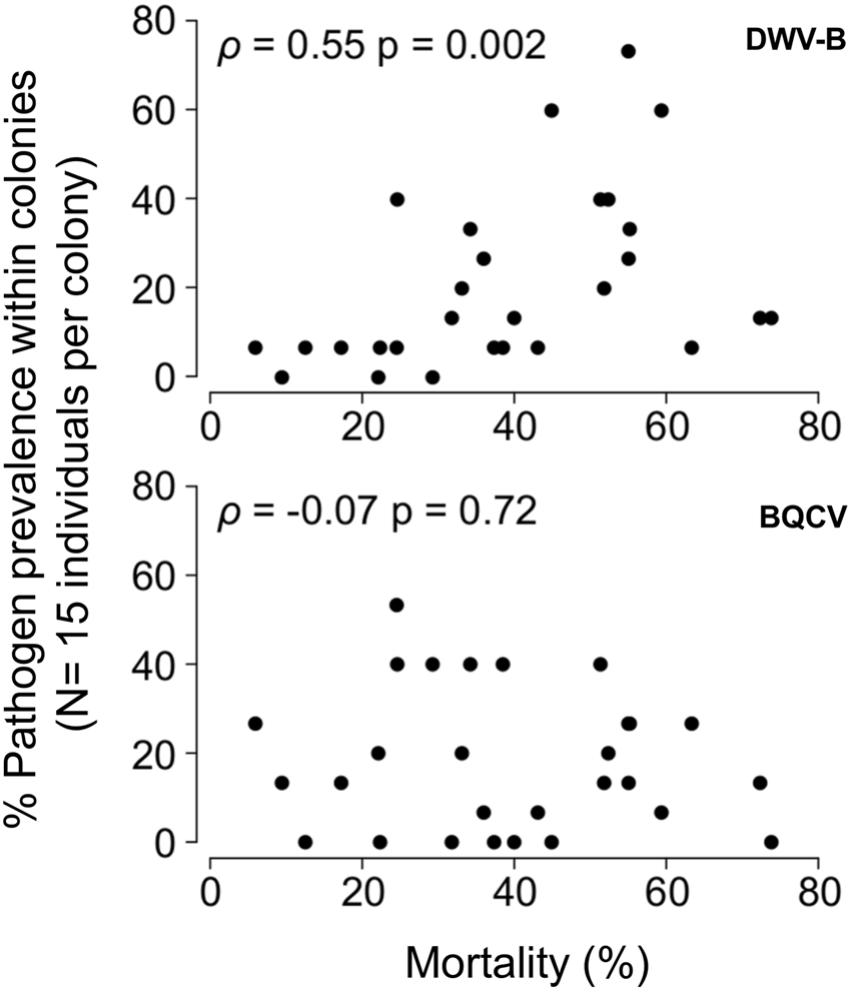
Prevalence of DWV-B and BQCV within colonies plotted against overwinter workforce mortality. Each point represents the prevalence of a virus in 15 individually MLPA-screened worker bees sampled from each colony in autumn. Spearman’s ρ and significance levels are shown for correlations.

**Figure 4:**
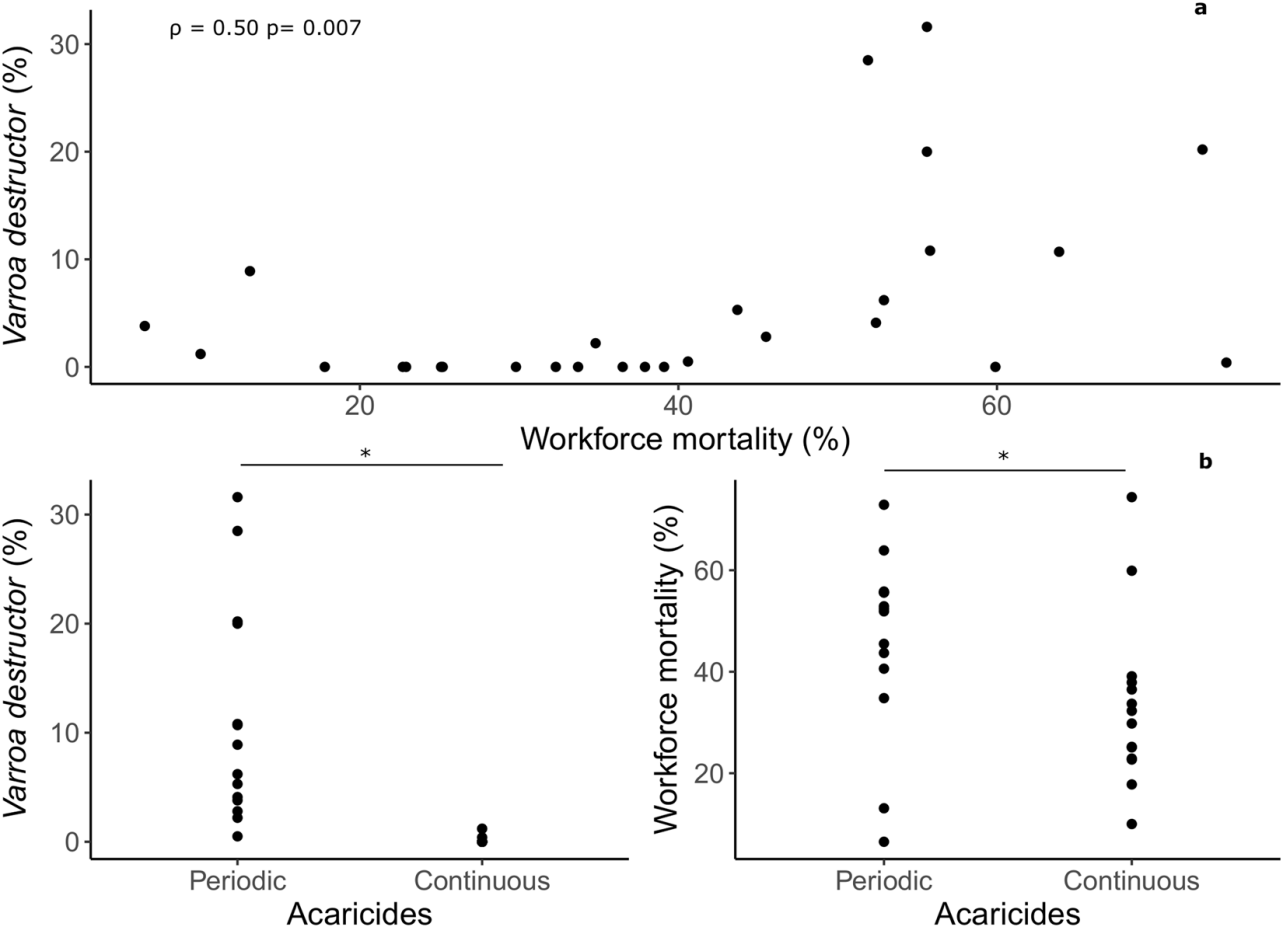
(**a**) Relationship between *V. destructor* infestation and overwinter mortality within each colony. Spearman’s ρ and significance levels are shown. (**b**) Levels of *V. destructor* infestation and overwinter mortality across the two acaricide treatments (in the periodic treatment, acaricides were removed from 26^th^ July until 18^th^ October 2011).

The relationships between pathogen prevalence and overwinter mortality for the DWV-B and BQCV viruses (Fig. 3), CBPV and Microsporidian pathogens (Supplementary Fig. S1), and *V. destructor* (Fig. 4) were tested using GLMs (Table 1) and Spearman’s correlations (Figs 3 and 4), which indicated that DWV-B and *V. destructor* were closely tied to overwinter colony decline but BQCV was not. In colonies suffering >50% adult worker mortality (N = 10 colonies), DWV-B prevalence and *V. destructor* infestation averaged (standard deviation, SD): 33% (SD 21%) and 13.3% (SD 11.3%) and in the remaining colonies (N = 18), 14.4% (SD 16.1%) and 1.4% (SD 2.5%) respectively.

*Varroa destructor* infestation and acaricide treatment were negatively associated (Wilcoxon signed rank test, *W = *195, p < 0.0001), demonstrating that intensive acaricide treatment had worked effectively to remove *V. destructor* mites from colonies (Fig. 4b). Additionally, *V. destructor* and within-colony DWV-B prevalence were positively correlated (GLM, t = 2.486, p = 0.02), while BQCV, CBPV and *Nosema* spp. were not found to be affected by the mite infestation levels or acaricide treatment (p > 0.05 in all cases).

### DWV composition

In order to verify the DWV-B variant detected in our study, a viral propagation derived from a DWV-B positive colony extract was subjected to ultra-deep sequencing. Results (presented in ref. 33) confirmed our qRT-PCR finding; DWV was the only virus detected in our field extract, with DWV-B being the main genetic variant detected. A very low background level of DWV-A was also present but accounted for only 0.13% of all DWV reads compared to 99.87% of DWV-B reads. A third DWV variant, DWV-C^38^, was not detected (Supplementary Table S3).

An important caveat is that, though we did not detect any DWV-A/B recombinants in our samples, either by qPCR or by ultra-deep sequencing, we cannot exclude the possibility that one or more colonies harboured a DWV-A/B recombinant that caused high mortality. If this is the case, then the recombinant comprises predominantly DWV-B at both 5′ (Lp gene) and 3′ (RdRp gene) parts of its genome and DWV-A somewhere in its centre.

## Discussion

Colony losses are a major source of ongoing honeybee population decline in temperate zones^21, 22, 39^. The *V. destructor* mite and its association with viruses have together been implicated as a possible cause of widespread losses of honeybee colonies globally^9, 11, 24, 26, 29, 40^. But determining which among several potential factors of OCL are more important has remained elusive. In a field study, we now find the prevalence of DWV-B, an emerging variant of DWV^17, 31, 32^, and *V. destructor* infestation to be highly correlated with overwinter honeybee worker loss. Low virus titres in bees surviving to spring further suggested that individuals infected with high DWV-B titres had died overwinter. To our knowledge, our study is the first to provide strong correlative evidence of this distinct genotypic variant of DWV and OCL in the absence of the classical DWV-A.

Colony losses have been linked to the presence of other positive ssRNA viruses, in particular to the ABPV-KBV-IAPV complex^11, 25^ and to SBPV^9^ that, in association with *V. destructor*, have been linked to high virulence and individual-level bee mortality^41, 42^. Our data are in line with the view that DWV, although less virulent when vectored by *V. destructor* than viruses such as ABPV and SBPV^40, 43^, may be more widespread and damaging at the colony and population level^44^. Unlike ABPV and SBPV, honeybee pupae (and their *V. destructor* ectoparasites) infected with DWV typically do not die prior to eclosion. Survival of infected pupae into adulthood permits the ongoing spread of infection by co-emerging *V. destructor* mites, and therefore increases the persistence of infections within colonies. Field evidence shows that, of the documented honeybee viruses, DWV occurs most frequently, and at high levels globally wherever *V. destructor* is also present^17^. In accordance, a recent study based on phylogenetic analysis of DWV strongly suggested that the dispersal of DWV around the globe followed a similar pattern to the spread of *V. destructor* mites^45^.

Although levels of *V. destructor* infestation in colonies were significantly correlated with the prevalence of DWV-B, making it difficult to disentangle their effects in OCL, reduced virus detection in the spring suggests that colony depopulation during winter can be attributed to the death of heavily DWV-B infected individuals. In addition, we have recently shown that, under lab conditions, DWV-B is more virulent that DWV-A, leading to significantly reduced honeybee worker survival even in the absence of *V. destructor* ^33^. Taken together, these results suggest that the variance in overwinter honeybee loss can be largely accounted for by differences among colonies in autumn DWV-B loads.

Interestingly, we only detected the DWV-B genotype among our honeybee samples, and found little evidence of either the classic DWV-A, or recombinant forms of DWV-A and -B. An increasing number of studies report the presence of recombinants between DWV-A /-B in nature^46–49^, and it has been suggested that recombinants may be more virulent than DWV-A or -B, at least in honeybee pupae^48^. In the present study, we did not detect any mixed infections in our field samples to indicate the presence of recombinants. In addition, recombinant DWV-A/B viruses so far reported by others^46–49^ all comprise DWV-A at their 3′ end (including the RdRp gene), whereas colonies in our study contained only DWV-B RdRp; we can therefore exclude the possibility that we oversaw previously detected recombinant variants. Yet we cannot exclude the possibility that one or more colonies in which we detected DWV-B may have harboured a novel recombinant DWV-A/B virus comprising DWV-B at its 5′ and 3′ parts, where respectively our Lp and RdRp primers bound to the DWV genome. Alternatively, for a small number of samples with low virus load, the Lp region remained uncharacterized (no amplification with either the DWV-A or DWV-B Lp primers), possibly due to the lower sensitivity of the Lp primers. Given a recent study by Dalmon *et al*.^49^, which suggests the presence of a recombination breakpoint located in the region where our Lp primers bind to the DWV genome, we cannot exclude the possibility of recombinants being present in some of our colonies.

Knowledge of the distribution of DWV genotypes across Europe and more widely across the globe is largely lacking. In a recent survey across Great Britain, we found that DWV-B is widespread across the landscape^33^. Preliminary data suggest that DWV-B in addition to recombinant DWV-A/-B forms are currently more prevalent in Europe than in North America^46, 48, 50^. Our findings highlight the significant risk posed by this emerging virus genotype to the wellbeing of managed honeybee populations; understanding the global distribution of DWV genotypes is an urgent necessity.

In conclusion, our findings support the view that DWV-B poses a particular problem in temperate zones, when queens cease egg laying during winter and the workforce is not replenished by newly emerging workers for several months. Given its putatively higher virulence, this specific genetic variant of DWV can be considered a sufficient cause of premature death in otherwise healthy adult worker bees, even in the absence of its vector the *V. destructor* mite. However, the extent and rate of overwinter decline of a standing population of honeybee workers in a colony, and therefore the chance of that colony’s survival into spring, will be a function of initial workforce size, virus load and prevalence, in addition to other factors such as availability of overwinter resources. Models of honeybee colony dynamics that incorporate *Varroa*-virus interactions explicitly (reviewed in ref. 51) seem especially appropriate in light of our data.

## Methods

### Field Experiment

Fourteen honeybee colonies from an apiary at Kenzingen (48°11′30″N 7°46′6″E) and 14 colonies at an apiary in Simonswald (48°6′1″N 8°3′21″E), both in Baden-Württemberg, Germany, were monitored overwinter from 2011–2012. All colonies were treated with the acaricides flumethrin (Bayvarol^®^) and coumaphos (CheckMite+^™^) except from 26^th^ July until 18^th^ October 2011, when acaricides was removed from half of the colonies at each site^47^ Worker bees from each colony were freeze-killed on dry-ice on 26^th^ September 2011 and 9^th^ April 2012 and subsequently preserved at −80 °C for pathogen analysis. To quantify levels of overwinter worker mortality we applied the Liebefeld method^52^ to estimate numbers of bees in each colony in autumn (17^th^ October 2011), after most brood rearing had stopped, and early spring (28^th^ February 2012), before new workers had enclosed. These data allowed us to quantify precisely overwinter mortality of each colony (Supplementary Table S2). Levels of *V. destructor* infestation were assessed by counting the number of mites associated with samples of 150 adult workers per colony (17^th^ October 2011)^53^ (Supplementary Table S2).

### RNA extraction and pathogen detection

For each colony, 30 adult worker bees from each sampling period (September 2011 and April 2012) were pooled in RNAse free mesh bags (Bioreba, Reinach, Switzerland) and crushed in 6 mL DEPC treated (RNAse free) water while snap-frozen in liquid nitrogen, 150 μl of which was used for RNA isolation. In addition, 15 adults per colony (collected during exactly the same sampling times as the pooled samples above) were individually crushed in 500 μl RLT buffer plus 1% b-mercaptoethanol, 100 μl of which was used for RNA isolation. RNA was obtained using the RNeasy mini kit in a QiaCube robot (Qiagen, Hilden, Germany). We thereby aimed to capture all RNA viruses in our extracts, including those with low titre.

We targeted the amplification of 10 widespread viruses and 2 Microsporidia (*N. ceranae*, *N. apis*). As we conducted an investigation of overwinter mortality when brood production largely ceases, we did not screen for brood diseases such as European and American Foulbrood (*Paenibacillus larvae*, *Melissococcus plutonius* respectively) or Chalkbrood (*Ascosphaera apis*). For virus screens of pooled and individual samples, we employed multiple ligation-dependent probe amplification (MLPA) using the RT-MLPA® kit (MRC-Holland, Netherlands) according to manufacturer’s instructions, using probes designed for the positive strand of the following RNA virus complexes: DWV-VDV-KV; CBPV; ABPV-IAPV-KBV; BQCV; SBPV; SBV, and β-actin (host housekeeping gene)^54^. Amplified fragments were viewed on a QIAxcel capillary fragment analyzer (Qiagen), using an acceptance threshold of 0.1 relative fluorescence units.

As MLPA probes were unable to distinguish between DWV-A and DWV-B, pooled samples were re-analyzed using qRT-PCR to gain higher resolution data on DWV and BQCV, the main RNA viruses found among colonies. Total cDNA was synthesized using M-MLV Revertase (Promega) following manufacturer’s instructions, using 800 ng of sample RNA. For absolute viral load, separate qPCRs were performed for DWV-A, DWV-B, BQCV and *A. mellifera* RP49 (housekeeping gene) in a Bio-Rad C1000 Thermal Cycler (Bio-Rad) using 2 x SensiMix SYBR and Fluorescein (Bioline), 0.2 µM of each primer and 1 µl of 1:10 diluted cDNA in a final volume of 10 µl. Negative controls comprising all reaction components except cDNA template were included in each run. Each reaction was performed in duplicate and the average quantification cycle (Ct) value was used (the maximum accepted Ct difference between replicates was set to one Ct). Amplification was performed using the following thermal profile: 5 min at 95 °C, followed by 40 cycles of 10 sec at 95 °C and 30 sec at 57 °C (including a read at each cycle). Post amplification melting curve analysis was used to check for non-specific amplification (50–90 °C with an increment of 0.5 °C s^−1^). To minimize risk of false positives, an upper threshold of 35 Ct for detection of DWV-A, DWV-B and BQCV was applied^55^.

For DWV-A/-B we conducted qRT-PCR amplification in two regions at either end of the genome: the leader polypeptide (Lp, 5′ end of genome) and RNA-dependent RNA polymerase (RdRp, 3′ end of genome), in order to screen for possible recombinants. Samples positive for DWV-B in both RdRp and Lp regions that showed negative amplification (or Ct > 35) for DWV-A (Lp and RpRd region) were classified as DWV-B positive. In the case of samples (n = 3 autumn, n = 5 spring samples) that showed positive amplification only in the DWV-B RdRp region and not in the DWV-B Lp region, samples were also considered to be DWV-B positive as no amplification was detected in the DWV-A Lp or DWV-A RdRp regions to indicate the presence of a recombinant. Absolute quantification of DWV-A, DWV-B and BQCV was calculated using duplicate DNA standard curves of purified PCR products (DWV-A/-B) or plasmids (BQCV, RP49), with efficiencies between 90% to 97% and correlation coefficients (R^2^) from 0.985–0.999. Pooled sample virus loads are reported as average viral titre per bee, calculated by the mean viral load of 30 pooled bees and taking into account the qRT-PCR starting quantities (Supplementary Table S2). Absolute quantities and prevalence of DWV-A and -B in pooled colony samples were calculated using the RdRp primers. Additionally, we also calculated normalized viral titres to the amount of RP49 mRNA per sample to account for differences in RNA integrity across samples (Supplementary Table S2). *Nosema* spp. detection was performed from total cDNA by RT-PCR using species-specific primers. All (q)RT-PCR primers used in the present study are listed in Supplementary Table S4. The high specificity of DWV primers spanning in the RdRp region has been previously verified in McMahon *et al*.^33^ (see Supplementary Table S3 in ref. 33).

### Virus verification

In order to verify the identity of DWV genotypes detected in our samples, one of the DWV-B positive pooled-colony samples, devoid of BQCV and CBPV, namely colony ts3, was used to produce a virus extract and was subsequently subjected to ultra-deep sequencing on an Illumina platform (GATC Biotech, Konstanz, Germany). Details of the method are available in McMahon *et al*.^33^, where exactly the same viral extract was used for experimental purposes.

### Statistical analysis

All analyses were performed in R v 3.1.3^56^. We compared pathogen presence in autumn and spring colonies using McNemar’s χ^2^ (repeated colony measure) and tested for differences in pathogen load in autumn and spring using paired Wilcoxon signed rank tests. The latter analysis was performed using either absolute viral titre derived from standard curves or viral titre normalized to the host housekeeping gene (RP49). As both approaches yielded the same results, reported test statistics refer to absolute viral titres. Bee mortality at apiaries was analyzed in a generalized linear model as a two-vector response variable (number of dying and surviving bees). *Varroa destructor* infestation and treatment were negatively associated but as *V. destructor* was a significantly better fit (in terms of residual deviance) than acaricide treatment, it was retained in the model. *Varroa destructor* infestation and DWV-B were positively associated (see results). While *V. destructor* was superior to DWV-B in terms of model fit, we conducted parallel models with either DWV-B (model 1) or *V. destructor* infestation (model 2) because the causal relationship between DWV-B or *V. destructor* and mortality is unclear. All quantitative predictors were standardized to a mean of zero and standard deviation of one prior to analysis.

Models containing either DWV-B or *V. destructor* in addition to BQCV, CBPV, *N. ceranae*, *N. apis* and apiary site were simplified by performing automated model selection using the ‘dredge’ function of the R-package ‘MuMIn’^57^. Inspection of residual deviance indicated overdispersion, which was accommodated by using a quasibinomial error structure. We used the Akaike information criterion adjusted for overdispersion and corrected for small sample sizes (QAICc^58^) calculated by the ‘dredge’ function for all plausible subsets of model, to select explanatory variables driving overwinter worker mortality. Model residuals were explored graphically, while Shapiro-Wilk’s and White’s Tests were used to examine normality (‘shapiro.test’ function) and heteroskedasticity (‘het.test’ function), respectively. McFadden’s pseudo *r* ^*2*^ values are reported. The Spearman’s rank correlation test was applied to assess the relationship between the prevalence of each pathogen (%) and overwinter worker mortality (%).

### Compliance with ethical standards

All experiments conducted and presented in the manuscript comply with the laws and rules of the institution and country in which they were performed.

### Data availability statement

The datasets generated during the current study are included in this published article (and its Supplementary Information files).

## Electronic supplementary material

Supplementary Information
