# Supplementary material for: The virulent, emerging genotype B of Deformed wing virus is closely linked to overwinter honeybee worker loss
Source: Sci Rep. 2017 Jul 12;7:5242. doi: 10.1038/s41598-017-05596-3 (PMC5507926; doi:10.1038/s41598-017-05596-3)
Supplement: Supplementary file 1 — Supplementary Information [file 41598_2017_5596_MOESM1_ESM.pdf]

Supplementary information: The virulent, emerging genotype B of *Deformed wing virus* is closely linked to overwinter honeybee worker loss

Myrsini E. Natsopoulou[1,†‡], Dino P. McMahon[2,3,4†], Vincent Doublet[1,5¤], Eva Frey[6], Peter Rosenkranz[6], Robert J. Paxton[1,2,5]

[1] Institute for Biology, Martin-Luther-University Halle-Wittenberg, Hoher Weg 8, 06120 Halle (Saale), Germany
[2] School of Biological Sciences, MBC, Queen's University Belfast, Belfast BT9 7BL, UK
[3] Institute of Biology, Free University Berlin, Schwendenerstr. 1, 14195 Berlin, Germany
[4] Department for Materials and Environment, BAM Federal Institute for Materials Research and Testing, Unter den Eichen 87, 12205 Berlin, Germany
[5] German Centre for Integrative Biodiversity Research Halle-Jena-Leipzig (iDiv), Deutscher Platz 5e, 04103 Leipzig, Germany
[6] Apicultural State Institute, University of Hohenheim, 70599 Stuttgart, Germany

†These authors contributed equally

‡Current address: Section for Organismal Biology, Department of Plant and Environmental Sciences, University of Copenhagen, Denmark

¤ Current address: Centre for Ecology and Conservation, University of Exeter, Penryn, UK

Table S1. Virus and host housekeeping gene (beta-Actin) presence/absence (MLPA) from pooled samples.

Colony	DWV complex		BQCV		CBPV		Actin	
	Autumn	Spring	Autumn	Spring	Autumn	Spring	Autumn	Spring
cs1	0	0	1	1	0	0	1	1
cs2	1	0	0	0	0	0	1	1
cs3	1	0	0	0	0	0	1	1
cs4	1	0	0	0	0	0	1	1
cs5	1	0	0	1	0	0	1	1
cs6	1	0	0	1	0	0	1	1
cs7	1	0	1	0	0	0	1	1
ck1	1	0	0	0	0	0	1	1
ck2	1	0	0	0	0	0	1	1
ck3	1	1	1	0	0	0	1	1
ck4	1	0	0	1	0	0	1	1
ck5	1	0	0	0	1	0	1	1
ck6	1	0	0	0	0	0	1	1
ck7	1	1	0	0	0	0	1	1
ts1	1	0	0	1	1	0	1	1
ts2	1	0	0	0	1	0	1	1
ts3	1	0	0	0	0	0	1	1
ts4	1	0	0	0	0	0	1	1
ts5	1	0	0	0	0	0	1	1
ts6	1	0	0	0	0	1	1	1
ts7	1	0	0	0	0	0	1	1
tk1	1	0	0	0	0	0	1	1
tk2	1	0	0	0	0	0	1	1
tk3	1	0	0	1	0	0	1	1
tk4	0	0	1	1	0	0	1	1
tk5	1	0	0	1	0	0	1	1
tk6	1	0	0	0	0	0	1	1
tk7	1	0	0	0	0	0	1	1

Table S2. A. Virus loads (qRT-PCR) from pooled samples as Ct values for viruses detected by MLPA (see Table S1). **B**. Ct values transformed into virus genome equivalents, averaged per bee, for those two viruses with Ct < 35, along with the normalized values relative to the absolute quantity of the host housekeeping gene RP49. **C.** Overwinter worker mortality (%) along with viral and *Nosema* spp. prevalence per colony. Pathogen prevalence was based on 15 individuals per colony while *V. destructor* infestation was estimated on a sample of 150 individuals per colony.

A.

Colony	DWV-B (RdRp)		DWV-B (Lp)		DWV-A (RdRp)		DWV-A (Lp)		BQCV (Cp)		RP49	
	Autumn	Spring	Autumn	Spring	Autumn	Spring	Autumn	Spring	Autumn	Spring	Autumn	Spring
cs1	34.79	na	na	na	***39.02***	***36.41***	na	na	32.73	33.02	35.96	34.43
cs2	22.06	na	33.76	na	***38.15***	na	na	na	***35.66***	***38.45***	31.48	30.14
cs3	19.21	na	28.44	na	***38.69***	na	na	na	35.35	na	34.08	31.97
cs4	22.03	na	35.02	na	***36.43***	na	na	na	***37.47***	na	34.72	29.47
cs5	28.39	na	***39.59***	na	***38.14***	na	na	na	***36.00***	***37.12***	32.83	27.96
cs6	17.19	na	29.17	na	***38.78***	***39.18***	na	na	32.51	na	34.61	35.76
cs7	22.01	na	32.21	na	***38.34***	na	na	na	19.38	na	28.04	28.90
ck1	21.21	34.04	31.07	na	***36.34***	na	na	na	32.22	na	29.46	32.16
ck2	20.13	32.14	28.90	na	***37.68***	***38.25***	na	na	31.16	***38.80***	28.45	30.95
ck3	19.87	20.24	28.78	32.77	na	***38.81***	na	na	28.13	34.40	33.46	28.18
ck4	23.06	34.57	32.00	na	***38.35***	na	na	na	33.49	33.22	30.79	30.84
ck5	20.45	28.82	35.48	na	***37.52***	na	na	na	31.24	***38.74***	32.49	31.78
ck6	21.09	na	31.19	na	na	na	na	na	***36.20***	***38.34***	34.76	32.62
ck7	26.34	na	34.98	***38.53***	na	na	na	na	31.45	***38.92***	33.04	29.57
ts1	30.12	na	33.03	na	***37.60***	na	na	na	35.21	na	30.31	34.30
ts2	29.67	na	***36.63***	na	***37.13***	na	na	na	34.79	na	31.03	33.99
ts3	22.5	na	30.73	na	na	na	na	na	***36.56***	na	33.03	32.69
ts4	22.94	na	31.57	na	na	***35.55***	na	na	34.93	***38.17***	33.51	32.61
ts5	20.78	na	27.78	na	***37.50***	na	na	na	**35.59**	na	34.22	35.49
ts6	18.44	na	28.53	na	***36.02***	***36.28***	na	na	35.40	na	30	35.34
ts7	21.54	na	29.51	na	***37.35***	na	na	na	30.93	na	28.1	33.31
tk1	20.6	na	28.39	na	na	na	na	na	27.20	***37.79***	34.45	31.03
tk2	22.7	na	31.81	na	na	***35.62***	na	na	32.92	na	33.2	30.79
tk3	18.75	na	28.82	na	***37.79***	na	na	na	32.15	***35.75***	30.46	33.88
tk4	***35.79***	na	na	na	***36.79***	na	na	na	27.65	34.68	31.57	31.69
tk5	20.81	na	30.44	na	***39.10***	na	na	na	29.81	33.84	34.5	32.30
tk6	21.02	34.47	29.82	na	***37.43***	***38.68***	na	na	31.83	***37.96***	34.2	32.17
tk7	21.24	na	30.05	na	na	na	na	na	33.27	na	32.97	32.95

c = control treated; t = pesticide treated; s = Simonswald; k = Kenzingen. Bold/italic Ct values > acceptance threshold (Ct = 35). na = not detected;
RdRp = RNA-dependent RNA polymerase; Lp = Leader polypeptide; Cp = Capsid protein

Table S2. Cont.

B.

Colony	DWV-B (RdRp)		BQCV (Cp)		DWV-B (RdRp) Normalized to RP49		BQCV (Cp) Normalized to RP49	
	Autumn	Spring	Autumn	Spring	Autumn	Spring	Autumn	Spring
cs1	301358		2E+06	2E+06	0.004509128		0.06355	0.01954
cs2	5E+08				0.896240745			
cs3	9E+09		335537		29.86662299		0.00319	
cs4	1E+09				7.35743038			
cs5	8E+06				0.036566367			
cs6	1E+10		1E+06		153.2693408		0.03099	
cs7	2E+09		2E+10		0.101596447		3.37688	
ck1	2E+09	31858	3E+06		0.422940383	1.54068E-05	0.00137	
ck2	2E+09	116216	3E+06		0.442219063	2.98097E-05	0.00148	
ck3	1E+10	3E+08	8E+07	428361	13.13280105	2.667018767	0.28887	0.00014
ck4	8E+08	21225	1E+06	2E+06	0.304060351	2.63357E-05	0.00136	0.00169
ck5	4E+09	908275	6E+06		4.839704616	0.004003648	0.01873	
ck6	2E+09				13.82242841			
ck7	8E+07		4E+06		0.156514321		0.02309	
ts1	8E+06		370572		0.002384866		0.00031	
ts2	5E+06		253119		0.00506875		0.00066	
ts3	1E+09				1.834678945			
ts4	8E+08		455627		1.879646072		0.00295	
ts5	3E+09				11.90782037			
ts6	1E+10		304670		3.546638523		0.00022	
ts7	1E+09		4E+06		0.142624919		0.00138	
tk1	3E+09		9E+07		15.50489468		1.02962	
tk2	1E+09		2E+06		1.803011696		0.00947	
tk3	1E+10		3E+06		3.914529268		0.00274	
tk4			6E+07	850053			0.11841	0.00108
tk5	3E+09		1E+07	1E+06	13.98768012		0.18009	0.00285
tk6	3E+09	20489	4E+06		10.06830247	0.000282625	0.03783	
tk7	2E+09		1E+06		3.967555442		0.00642	

Table S2. Cont.
C.

Colony	overwinter worker mortality (%)	Varroa infestation (%)	DWV-B prevalence (%)	BQCV prevalence (%)	CBPV prevalence (%)	*N. ceranae* prevalence (%)	*N. apis* prevalence (%)
		Autumn	Autumn	Autumn	Autumn	Autumn	Autumn
cs1	43.66	5.3	6.7	6.7	0	0	0
cs2	6.51	3.8	6.7	26.7	0	0	0
cs3	34.78	2.2	33.3	40	0	6.7	0
cs4	52.93	6.2	40	20	6.7	0	0
cs5	45.47	2.8	60	0	0	0	0
cs6	13.08	8.9	6.7	0	6.7	0	0
cs7	40.55	0.5	13.3	0	6.7	0	0
ck1	55.57	31.6	73.3	26.7	0	6.7	0
ck2	55.6	20	26.7	13.3	0	0	0
ck3	52.38	4.1	20	13.3	0	6.7	0
ck4	72.9	20.2	13.3	13.3	0	0	0
ck5	55.75	10.8	33.3	26.7	6.7	0	0
ck6	51.9	28.5	40	40	0	13.3	0
ck7	63.9	10.7	6.7	26.7	6.7	0	0
ts1	22.91	0	6.7	0	6.7	6.7	6.7
ts2	33.67	0	20	20	13.3	0	0
ts3	32.34	0	13.3	0	13.3	6.7	0
ts4	74.38	0.4	13.3	0	0	0	0
ts5	59.92	0	60	6.7	0	0	0
ts6	36.54	0	26.7	6.7	0	0	0
ts7	37.91	0	6.7	0	0	0	0
tk1	25.17	0	40	40	0	6.7	0
tk2	17.79	0	6.7	13.3	0	0	0
tk3	25.09	0	6.7	53.3	0	0	0
tk4	22.68	0	0	20	0	0	0
tk5	39.06	0	6.7	40	0	0	0
tk6	29.82	0	0	40	0	0	6.7
tk7	10	1.2	0	13.3	0	0	0

Table S3. Genome of origin for Illumina sequence reads from the DWV-B field extract (from McMahon et al.[1]).

Origin	DWV-B reads
Apis	15,276,447 *(74.5%)*
Viruses	
DWV-B	4,547,491 *(22.2%)*
DWV-A	6,157 *(0.03%)*
Bacteria	
	1,011 *(0.0049%)*
Overhead	
phiX	26,355 *(0.13%)*
sequencing adapters	3,555 *(0.017%)*
low complexity	602,276 *(2.9%)*
Unknown	47,587 *(0.23%)*
Total	20,510,879

Table S4. Primers used in this study. The position of DWV-A and DWV-B primers across the genome are indicated in brackets (Accession number in Genbank: DWV-A ref: NC_004830; DWV-B ref: NC_006494). Minimum detection limit is based upon standard curves of viral dilutions run simultaneously with the samples.

Primer Target	Name	Sequence	Application	Amplicon size (bp)	Reference	Minimum detection limit (ct=35) in viral genome equivalents per reaction
DWV-A (Lp)	DWV-F1425	CGTCGGCCTATCAAAG	qPCR	417 position[1415...1831]	[2]	~1.7x 10^3
DWV-A (Lp)	DWV-B1806	CTTTTCTAATTCAACTTCACC	qPCR		"	
DWV-B (Lp)	VDV1-F1409	GCCCTGTTCAAGAACATG	qPCR	413 position [1392...1804]	"	~1.5x10^3
DWV-B (Lp)	DWV-B1806	CTTTTCTAATTCAACTTCACC	qPCR		"	
DWV-A (RdRp)	DWV-F2	TGTCTTCATTAAAGCCACCTGGAA	qPCR	140 position [8646...8785]	[1]	~4.9
DWV-A (RdRp)	DWV-R2a	TTTCCTCATTAACTGTGTCGTTGAT	qPCR		"	
DWV-B (RdRp)	VDV-F2	TATCTTCATTAAAACCGCCAGGCT	qPCR	140 position [8619...8758]	"	~11.33
DWV-B (RdRp)	VDV-R2a	CTTCCTCATTAACTGAGTTGTTGTC	qPCR		"	
BQCV	BQCV-F7893	AGTGGCGGAGATGTATGC	qPCR	294	[2]	~3x10^2
BQCV	BQCV-B8150	GGAGGTGAAGTGGCTATATC	qPCR		"	
RP49	RP49-qF	AAGTTCATTCGTCACCAGAG	qPCR	205	[3]	~3.3x10^4
RP49	RP49-qB	CTTCCAGTTCCTTGACATTATG	qPCR		"	
N. ceranae	Ncer-F	CAATATTTTATTATTTTGAGAGA	RT-PCR	232	[4]	NA
N. ceranae	Ncer-R	TATATTTATTGTATTGCGCGTGCA	RT-PCR		"	
N. apis	Napis-F	CAATATTTTATTGTTCTGCGAGG	RT-PCR	208	"	NA
N. apis	Napis-R	AAAGTCTATTGTATTGCGCGTGCT	RT-PCR		modified from [4]	

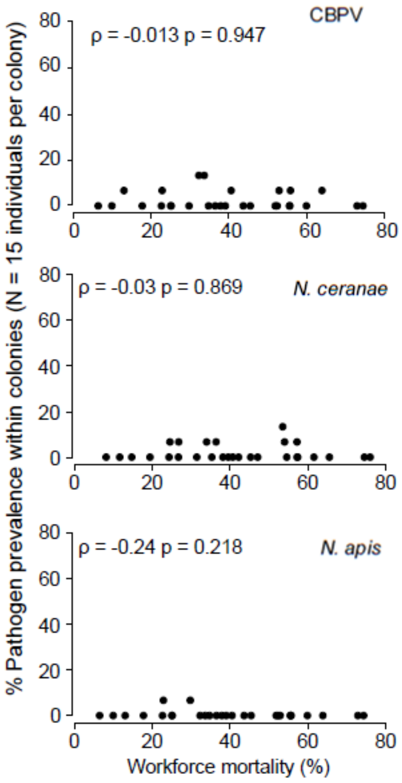

Figure S1. Relationship between CBPV, *N. ceranae* and *N. apis* prevalence within colonies in autumn (estimated from 15 individually screened honeybees) and overwinter mortality. Spearman's ρ and significance levels are shown.

References

1. McMahon, D. P. *et al.* Elevated virulence of an emerging viral genotype as a driver of honeybee loss. *Proc. R. Soc. B Biol. Sci.* (2016). doi:10.1098/rspb.2016.0811
2. Locke, B., Forsgren, E., Fries, I. & de Miranda, J. R. Acaricide treatment affects viral dynamics in *Varroa destructor*-infested honey bee colonies via both host physiology and mite control. *Appl. Environ. Microbiol.* **78,** 227–235 (2012).
3. Yañez, O. *et al.* Deformed wing virus and drone mating flights in the honey bee (*Apis mellifera*): Implications for sexual transmission of a major honey bee virus. *Apidologie* **43,** 17–30 (2012).
4. VanEngelsdorp, D. *et al.* Colony collapse disorder: a descriptive study. *PLoS One* **4,** e6481 (2009).
